# Supplementary material for: Nonalcoholic Fatty Liver Disease Induced by High-Fat Diet in C57bl/6 Models
Source: Nutrients. 2019 Dec 16;11(12):3067. doi: 10.3390/nu11123067 (PMC6949901; doi:10.3390/nu11123067)
Supplement: Supplementary file 1 [file nutrients-11-03067-s001.pdf]

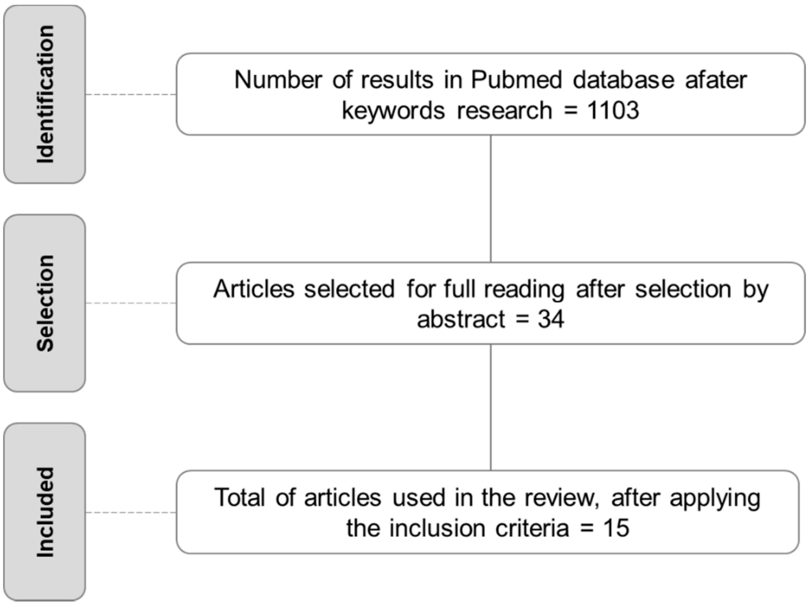

Supplementary Figure 1. Flowchart of the methodology used to choose the articles that comprised this review
